# Metabolic regulation to treat bipolar depression: mechanisms and targeting by trimetazidine

**DOI:** 10.1038/s41380-023-02134-8

**Published:** 2023-06-29

**Authors:** Sourav Khanra, Preethi Reddy, Anna Giménez-Palomo, Chun Hui J. Park, Bruna Panizzutti, Madeleine McCallum, Shyam Sundar Arumugham, Shreekantiah Umesh, Monojit Debnath, Basudeb Das, Ganesan Venkatasubramanian, Melanie Ashton, Alyna Turner, Olivia M. Dean, Ken Walder, Eduard Vieta, Lakshmi N. Yatham, Isabella Pacchiarotti, Y. C. Janardhan Reddy, Nishant Goyal, Muralidharan Kesavan, Lluc Colomer, Michael Berk, Jee Hyun Kim

**Affiliations:** 1 Department of Psychiatry, Central Institute of Psychiatry, Ranchi, Jharkhand, India; 2 Department of Psychiatry, National Institute of Mental Health and Neuro Sciences (NIMHANS), Bengaluru, Karnataka India; 3 Bipolar and Depressive Disorders Unit, Hospital Clínic, University of Barcelona, Institut d’Investigacions Biomèdiques August Pi i Sunyer (IDIBAPS), Mental Health Biomedical Research Networking Center (CIBERSAM), Madrid, Spain; 4 IMPACT, The Institute for Mental and Physical Health and Clinical Translation, School of Medicine, Deakin University, Geelong, VIC Australia; 5 Department of Human Genetics, NIMHANS, Bengaluru, Karnataka India; 6 Florey Institute of Neuroscience and Mental Health, University of Melbourne, Parkville, VIC Australia; 7 Department of Psychiatry, University of British Columbia, Vancouver, BC Canada

**Keywords:** Bipolar disorder, Drug discovery

## Abstract

Bipolar disorder’s core feature is the pathological disturbances in mood, often accompanied by disrupted thinking and behavior. Its complex and heterogeneous etiology implies that a range of inherited and environmental factors are involved. This heterogeneity and poorly understood neurobiology pose significant challenges to existing drug development paradigms, resulting in scarce treatment options, especially for bipolar depression. Therefore, novel approaches are needed to discover new treatment options. In this review, we first highlight the main molecular mechanisms known to be associated with bipolar depression–mitochondrial dysfunction, inflammation and oxidative stress. We then examine the available literature for the effects of trimetazidine in said alterations. Trimetazidine was identified without a priori hypothesis using a gene-expression signature for the effects of a combination of drugs used to treat bipolar disorder and screening a library of off-patent drugs in cultured human neuronal-like cells. Trimetazidine is used to treat angina pectoris for its cytoprotective and metabolic effects (improved glucose utilization for energy production). The preclinical and clinical literature strongly support trimetazidine’s potential to treat bipolar depression, having anti-inflammatory and antioxidant properties while normalizing mitochondrial function only when it is compromised. Further, trimetazidine’s demonstrated safety and tolerability provide a strong rationale for clinical trials to test its efficacy to treat bipolar depression that could fast-track its repurposing to address such an unmet need as bipolar depression.

## Introduction

Bipolar disorder (BD) is characterized by recurring manic or hypomanic episodes that alternate with depressive episodes [1–3]. Manic episodes include symptoms such as increased energy and activity, elevated mood, disinhibition, irritability, and psychotic symptoms. Depressive episodes are defined by decreased energy, fatigue, pervasive sadness, suicidal thoughts, and cognitive difficulties.

BD is a top 10 cause of disability that has threadbare treatment options compared to any other major cause of disability [4]. Its global disability-adjusted life years have worsened from 6 to 9 million in the last 3 decades [5]. This disability is largely driven by bipolar depression [4, 6]. Only four evidence-based monotherapies approved by the FDA are available for treating acute bipolar depression [7]. Despite treatment, people with BD spend >70% of their symptomatic periods depressed [4, 6]. BD also has higher suicide/mortality rates than any other psychiatric disorder [4]. This has remained unchanged for decades [5] and reflects a continued failure in developing effective therapies for bipolar depression [8, 9].

An important step towards treating bipolar depression is differentiating unipolar and bipolar depression [10–12]. Antidepressant monotherapy is not recommended to treat bipolar depression [7, 13], highlighting the need to understand the biological mechanisms specific to bipolar depression. The failure in bipolar depression treatment discovery is largely due to its complex pathophysiology with many known and unknown biological and environmental factors [4]. Typical drug development paradigms that target single proteins insufficiently address this challenge [14–17]. We have bypassed this obstacle using a novel cross-disciplinary adaptation of an in silico treatment discovery model without any a priori hypothesis [18], which identified trimetazidine as having transcriptomic effects that mimic a combination of first-line BD medications [19]. Importantly, trimetazidine’s main mechanism of action in boosting mitochondrial energy generation when mitochondrial function is impaired dovetails with previous research highlighting bipolar depression as a state of decreased mitochondrial energy generation [20–25]. Our discovery is critical – there currently are no psychiatric medications that directly target mitochondrial dysfunction.

**Fig. 1:**
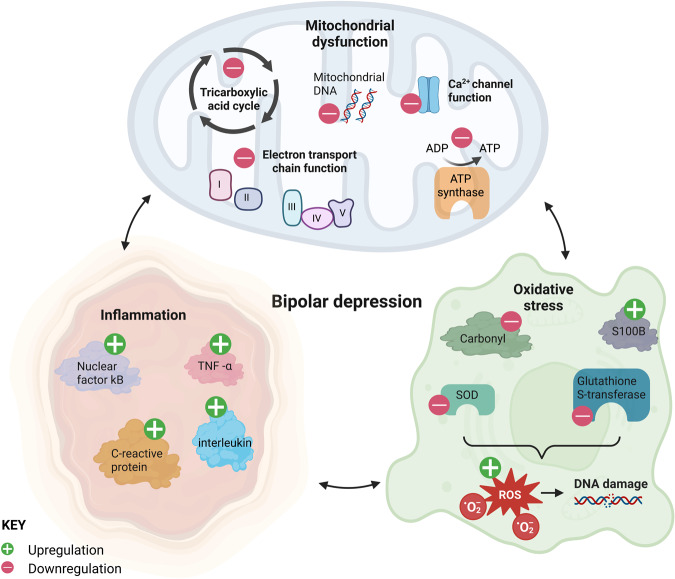
Bipolar depression is consistently associated with mitochondrial dysfunction, inflammation, and oxidative stress. These three major processes are closely connected with each other. For example, reduced expression of mitochondrial electron transport chain complexes I-V leads to inefficient oxidation of glucose and/or fatty acids. This can impair adenosine diphosphate (ADP) to adenosine triphosphate (ATP) conversion. In bipolar depression, increased calcium binding protein S100B has been observed, coupled with decreased superoxide dismutase (SOD), glutathione S-tranferase and carbonyl. These alterations can exacerbate inefficient oxidation and vice versa, and increase reactive oxygen species (ROS) overall to damage DNA. Chronic bipolar depression is associated with increased peripheral and central inflammation indicated by biomarkers such as elevated tumor necrosis factor alpha (TNF-α). Inflammation is more often observed in chronic rather than acute bipolar disorder, suggesting that it may be the result of prolonged mitochondrial dysfunction and oxidative stress. Figure created with Biorender.com.

Trimetazidine is being assessed for its efficacy in treating bipolar depression in an international multi-site clinical trial (Australian New Zealand Clinical Trials Registry registration: ACTRN12622000474752). Notably, trimetazidine’s potential to treat bipolar depression goes beyond addressing mitochondrial dysfunction to reversing inflammation and oxidative stress [26–29]. The present review will first focus on these inter-related biological mechanisms underlying bipolar depression (Fig. 1) and then highlight trimetazidine’s potential application in its treatment. While previous reviews examine BD overall with the described studies predominantly focused on the mania phase of BD, our aim here is to navigate biological processes that are strongly associated with bipolar depression.

## Mitochondrial dysfunction in bipolar depression

There has been a recent conceptualization of BD as a mitochondrial disorder, with the suggestion that energy production is increased in mania and decreased in the depressive phase [30, 31]. The brain is the body’s major consumer of the primary energy molecule adenosine triphosphate (ATP) [32]. ATP production and mitochondrial respiration in the brain may be increased in bipolar mania, while mitochondrial function and brain metabolism may be reduced in the depressive or euthymic phases of BD [33–36]. Accordingly, the disorder is now thought of as a failure in the regulation of mitochondrial energy generation [20]. For example, mitochondria in the postmortem prefrontal cortex of people with BD were found to exhibit smaller size and tendency to concentrate more within the perinuclear region compared with people without BD [37]. These differences were not related to lithium exposure [37], suggesting that mitochondrial abnormalities are not caused by BD treatment. Considering that people with BD spend >70% of their symptomatic periods depressed [4, 6], the smaller mitochondria likely suggest reduced function related to bipolar depression. In the past two decades, high quality genetic, transcriptomic, protein, enzymatic, and pharmacotherapeutic studies have consistently highlighted reduced mitochondrial function associated with bipolar depression.

### DNA evidence

Genetic studies support reduced mitochondrial function in chronic BD, which may relate to its dominant phase of bipolar depression. Clinically stable patients with BD showed significantly lower leukocyte mitochondrial DNA copy number and higher mitochondrial oxidative damage compared with participants without any psychiatric illness [38]. Several mutations in mitochondrial DNA sequence have been found in patients with BD, with several nonsynonymous nucleotide substitutions found in genes encoding complex I subunits [39]. These lead to reduced complex I activity and impaired mitochondrial calcium uptake [40]. Elevated intracellular calcium, in particular monoamine regulated calcium signaling, is one of the most consistent findings in the disorder [41, 42]. Further, the calcium/calmodulin-activated kinase kinase 2 (*CAMKK2*) gene single nucleotide polymorphism (SNP) rs1063843 is associated with an increased risk of BD [43]. *CAMKK2* is a gene regulating mitochondrial function, and when deleted, cellular respiration is reduced [44]. *CAMKK2* rs1063843 is associated with reduced CAMKK2 mRNA expression [45], consistent with the idea of reduced mitochondrial function and energy generation in bipolar depression.

### RNA, protein, and enzymatic evidence

A global downregulation of the expression of genes encoding mitochondrial proteins, such as those encoding mitochondrial electron transport chain (ETC) components for ATP production, has been reported in postmortem prefrontal cortex samples of BD patients receiving pharmacological treatment [46]. Downregulations in gene-expression profiles of ETC complexes I, IV and V was also reported in the postmortem frontal cortex of people with BD compared to control samples without BD [47]. Similarly, protein and enzymatic assessments of ETC complexes in the postmortem prefrontal cortex showed that levels of the ETC complex I subunit and complex I activity were decreased significantly in patients with BD compared with nonpsychiatric individuals [48]. These findings all indicate reduced brain ATP production in chronic BD.

Peripheral tissue assays of ETC gene expression and complex activity are generally supportive of reduced mitochondrial function in bipolar depression. In whole blood of people with BD in the depressive phase, the expression of genes in the ETC pathway are the most significantly altered compared to healthy controls, with 22 genes upregulated and 2 downregulated in BD [49]. Recent enzymatic analyses in lymphocytes showed that BD patients in the depressive phase had lower levels of mitochondrial complex II activity compared to those in the euthymic phase, with a significant negative correlation between the Hamilton Depression Rating Scale score and mitochondrial complex II activity [50], providing a strong link between reduced mitochondrial function and current bipolar depressive phase. However, one study showed that ETC complex activity in the peripheral blood mononuclear cells was not different between euthymic patients with BD compared to healthy participants [51].

The tricarboxylic acid cycle produces key substrates used by the mitochondrial ETC for ATP production [52]. A postmortem study reported reduced neural mRNA expression of the tricarboxylic acid cycle enzyme malate dehydrogenase in BD [53]. However, tricarboxylic acid enzymatic activity in leukocytes of recent-onset drug-naïve people with BD in a depressive episode was not different from healthy participants [52]. These findings suggest tricarboxylic acid cycle changes may be a consequence of chronic BD and/or are specific to tissue.

Some evidence involving calcium homeostasis and apoptotic proteins in mitochondrial dysfunction has been reported in bipolar depression. The anti-apoptotic protein Bcl-2, encoded in a putative BD susceptibility gene locus, modulates endoplasmic reticulum-calcium dynamics that is related to mitochondrial function [54]. In B lymphoblast cell lines derived from blood samples in people with BD, Bcl-2 SNP rs956572 (G/G) genotype was associated with the low Bcl-2 mRNA and protein levels compared to healthy control cell lines [55]. The Bcl-2 SNP rs956572 (G/G) genotype also showed higher basal intracellular calcium concentrations compared with other genotypes and with healthy individuals [55]. When the expression of 44 pro-apoptotic genes was assessed in the postmortem hippocampus, 19 of those genes were significantly upregulated in BD, which was unrelated to the exposure to mood stabilizers [56]. Such upregulation was not observed in schizophrenia [56], suggesting that the upregulation of apoptotic gene expression is unlikely to be related to mania but may subserve the dominant phase of bipolar depression. A postmortem frontal cortex study also showed significant increases in protein and mRNA levels of pro-apoptotic factors and significant decreases in levels of anti-apoptotic factors in BD compared to nonpsychiatric controls [54].

There is a link between apoptotic markers and mitochondrial proteins, with a significant negative correlation between mitochondrial fission/fusion proteins and apoptotic markers in peripheral blood cells of people with BD [57]. The levels of pro-apoptotic active caspase-3 protein were significantly increased while the anti-apoptotic proteins and mitochondrial fusion-related proteins were significantly decreased in BD patients compared to healthy controls [57]. A positive correlation between mitochondrial fusion-related proteins with mitochondrial content markers was also reported [57]. In that study, younger onset of bipolar depression (average 15.31 years of age) compared to mania (average 19.11 years of age) in BD patients was reported, suggesting that the findings likely relate to bipolar depression rather than mania. The inverse relationship between apoptosis and mitochondrial function in BD highlights reduced mitochondrial function with increased apoptosis in bipolar depression.

### Pharmacotherapeutic evidence

Postmortem frontal cortex analyses showed higher ETC complex I in people with BD receiving lithium than those not receiving lithium [47]. Long-term treatment of cultured cells with lithium and valproate also enhanced cellular respiration rate and mitochondrial function as determined by mitochondrial membrane potential and oxidation [58]. Lithium and valproate also protect against mitochondria-mediated cell toxicity [58]. This may suggest that increased mitochondrial function likely plays a role in mediating the therapeutic effects of lithium and valproate treatments in BD.

Consistent with this idea, agents that are known to enhance mitochondrial function, known as mitochondrial modulators, have been studied as potential adjunct treatments for bipolar depression [59]. N-acetylcysteine, which amongst many other effects, enhances the efficiency of mitochondrial energy generation has an efficacy signal in some but not all trials examining bipolar depression [60, 61]. Coenzyme Q10 is a lipid-soluble benzoquinone present in the phospholipid bilayers of mitochondria that has two main roles: it shuttles electrons within the mitochondrial ETC and serves as a potent antioxidant [62]. Coenzyme Q10 significantly decreased bipolar depression severity without changing creatine kinase activity [63]. Creatine plays a role in brain energy homeostasis, acting as a buffer for cytosolic and mitochondrial pools of the cellular energy currency ATP. Creatine monohydrate as adjunctive treatment in people with bipolar depression showed a significant improvement in verbal fluency compared with placebo, but not on other neuropsychological tests [64]. Another trial did not find creatine monohydrate to be efficacious compared to placebo in treating bipolar depression, but creatine monohydrate performed better than placebo when remission criteria were considered [65]. However, hypomania/mania switch has also been reported with creatine monohydrate supplementation [65, 66], highlighting that targeting only mitochondrial function may not be sufficient to treat bipolar depression.

## Inflammation in bipolar depression

Cytokines and other inflammatory markers suppress mitochondrial energy generation. Inflammatory mechanisms may play a crucial role in BD pathophysiology via their regulation of synaptic transmission/plasticity and neuronal survival [67, 68]. The evidence has been largely RNA- and protein-based with increased concentrations of interleukin (IL)-4, tumor necrosis factor (TNF)-*α*, soluble TNF receptor 1 (sTNFR1), and soluble IL-2 receptor consistently reported in BD compared to healthy controls [67, 68]. However, clarity is needed to interpret these findings in the context of depressive vs manic phases.

### RNA and protein evidence

In blood, a combination of mRNA levels of inflammation genes has been shown to differentiate people with BD in the euthymic phase from healthy controls with diagnostic power of 0.85 [69]. While evidence of shared aberrant expression of inflammatory genes in people with BD and their offspring suggests inflammation as a risk factor [70], a twin study showed that shared environmental factors dominate more than the genetic factors in the shared gene expression [71]. That is, inflammation in BD likely reflects a consequence of the disease.

Seven depressed and one manic BD patients showed higher inflammatory gene expression compared to healthy controls and euthymic BD patients, who did not differ from each other [72]. Another study reported BD patients (75 euthymic, 14 manic/hypomanic, 45 depressive) had higher levels of all cytokines, including slL-2R, C-reactive protein (CRP) and sTNFR1 than the healthy controls [73], with depressed BD patients having reduced sTNFRl and slL-2R compared to those in mania and euthymia [73]. A meta-analysis indicated that increased TNF-α levels may be present in both mania and depression in BD, while increased sTNfRI and CRP may be specific for mania [74]. Despite some inconsistencies in the results, especially regarding the different mood states of BD [75], these findings support the potential of anti-inflammatory medications to treat BD without the risk of switching between the mood states.

### Pharmacotherapeutic evidence

Baseline blood IL-6 levels in people currently experiencing bipolar depression inversely predict antidepressant efficacy of sleep deprivation and sleep phase advance [76]. That is, higher inflammation was associated with reduced therapeutic efficacy [76]. Similarly, BD patients who are lithium responders showed significantly lower levels of inflammatory markers such as IL-2, IL-6, and IL-10 compared to non-responders [77].

Several clinical and preclinical studies have shown that the mechanism of action of mood stabilizers may include reduction of inflammatory cytokines [78]. BD patients who started pharmacotherapy for the first time showed decreased cytokine production after 3 months of lithium treatment [77]. In addition, monotherapy or polytherapy with lithium, carbamazepine, valproate, and/or antipsychotics in people with BD (mainly euthymic) was associated with downregulated expression of inflammatory genes [70]. However, antidepressants, benzodiazepines, and levothyroxine medications were not associated with changes in inflammatory gene expression [70]. A systematic review of monotherapies reported that long-term use of lithium and euthymia was associated with normal cytokine levels [79]. Valproate use was not associated with levels of cytokines, but only two studies met the monotherapy criteria [79]. A pilot trial on the efficacy of interpersonal social rhythm therapy with quetiapine or placebo in patients experiencing bipolar depression reported that the quetiapine group had higher pro-inflammatory and lower anti-inflammatory cytokines compared to the placebo group [80]. However, this pilot trial did not match baseline cytokine levels between the pharmacotherapy conditions nor report any changes in depression levels at the end of treatment, hence the results may not be related to bipolar depression symptomology.

## Oxidative stress in bipolar depression

Mitochondrial dysfunction and inflammation can lead to oxidative stress. Oxidative stress itself suppresses mitochondrial function, which contributes to diminished neuroplasticity and neurogenesis, with increased apoptosis and neurodegeneration in BD [23, 25, 81]. Indeed, increased oxidative stress evidenced by DNA, RNA, protein, and enzymatic analyses has been consistently reported in bipolar depression [82–85]. Pharmacotherapeutic evidence also supports the association between bipolar depression treatment efficacy and antioxidant activity [86].

### DNA evidence

SNPs of the antioxidant genes superoxide dismutase 2 (SOD2) and glutathione peroxidase 3 were differentially associated with brain volumes in depressed youth with BD [82]. Specifically, there was smaller anterior cingulate cortex in the BD SOD2 rs4880 GG group compared to the healthy group with the same SNP, and smaller frontal and parietal lobes in the BD glutathione peroxidase 3 rs3792797 A-allele carrier group compared to the BD CC and HC A-allele carrier groups. SOD rs4880 was associated with increased reactive oxygen species (ROS) [87, 88], which may play a role in the reduced brain volume. Glutathione peroxidase 3 rs3792797 is associated with increased risk for Crohn’s Disease via reduced antioxidant pathways [89]. In addition, leukocytes from euthymic BD patients have increased oxidative stress-induced DNA damage and decreased base excision repair capacity than healthy individuals [90]. Overall, these oxidative stress findings may be related to the prolonged periods of depression in BD.

### RNA, protein, and enzymatic evidence

In the serum of 30 patients with bipolar depression, oxidant nitric oxide levels were increased while antioxidant SOD levels were decreased compared to healthy controls, suggesting that the ability to cope with oxidative stress is impaired in bipolar depression [83]. Consistent with this finding, postmortem studies have reported markedly downregulated gene expression of antioxidant enzymes such as SOD1 and glutathione S-transferase in the hippocampus of BD patients, a finding that may relate to bipolar depression as the dominant phase [56]. Glutathione S-transferase conjugates glutathione, the major antioxidant in brain, to form nontoxic products [91]. Increased serum thiobarbituric acid reactive substances and decreased Na+–K+-ATPase activity are indicative of oxidative stress such as lipid peroxidation [92], which are observed in unmedicated patients with bipolar depression compared to healthy controls [84].

People with bipolar depression also show more oxidative protein damage measured by increased serum protein carbonyl content over and above people with mania or euthymia and healthy controls [85]. There was a correlation between decreased complex I activity and increased protein oxidation (measured by protein carbonylation, and levels of 3-nitrityrosine) when investigating postmortem prefrontal cortex brains from BD affected and non-affected individuals [48]. Additionally, a negative correlation between complex II activity and oxidative stress measures has been reported in BD patients during depressive episodes, suggesting that mitochondrial oxidative stress related mitochondrial dysfunction may contribute to bipolar depression [50]. Calcium binding protein S100B is a measure of accumulated oxidative stress, and its level in individuals with bipolar depression is approximately two-fold higher compared to healthy subjects [93]. In these participants, S100B levels correlated with cytochrome c release, a mitochondrial apoptotic marker [93], supporting the oxidative stress/mitochondrial dysfunction interplay in bipolar depression.

### Pharmacotherapeutic evidence

Serum thiobarbituric acid reactive substances were decreased by lithium in a clinical trial in bipolar depression, with a further decrease observed in lithium responders compared to non-responders [86]. These findings are consistent with preclinical results in which chronic lithium treatment alleviates oxidative stress induced by chronic variable stressors (e.g., restraint and noise) in rats showing depression-like symptoms via increasing SOD and total antioxidant activity [94]. Lithium has also demonstrated antioxidant effects by increasing mRNA expression and protein levels of different glutathione S-transferase isoenzymes in rat cortical cells [95]. In addition, lithium or valproate significantly inhibit oxidative damage to lipids and proteins induced by various insults in rat cerebral cortical cells [96]. Lithium and valproate have also been shown to inhibit H_2_O_2_-induced and complex I inhibitor rotenone-induced cytochrome c release, caspase-3 activation and cell death in human neuroblastoma cells and in murine hippocampal cells [97]. In these cells, lithium, valproate, carbamazepine and lamotrigine increased the levels of the major antioxidant glutathione and the expression of glutamate-cysteine ligase, the rate-limiting enzyme in glutathione synthesis [97]. These results support the neuroprotective function of mood stabilizing drugs against oxidative stress.

A double-blind randomized trial of adjunctive N-acetylcysteine, a precursor of glutathione, in individuals with bipolar depression yielded positive results compared to placebo [98, 99]. N-acetylcysteine as a maintenance treatment for bipolar depression also appeared to be beneficial [100]. These promising results from Australia potentiated more trials of N-acetylcysteine in Brazil, Denmark and USA to treat bipolar depression [101–106]. A recent meta-analysis of all the double-blind, placebo-controlled, randomized clinical trials of N-acetylcysteine as adjunctive therapy in bipolar depression confirmed its superiority over placebo in reducing depressive symptoms with a moderate effect size (95% confidence interval 0.06–0.84) [61]. While these findings are highly promising, the substantial heterogeneity (*I*^2^ = 49%) reflect more recent trial outcomes which have not shown statistically significant N-acetylcysteine and placebo differences. Moderating analyses of baseline depression scores, mean N-acetylcysteine dose and duration of study did not explain the heterogeneity [61, 104–106].

## Trimetazidine

Treatment options for bipolar depression are scarce. It is an urgent imperative to identify drugs that can target the biological processes associated with bipolar depression to maximize the chance of positive outcomes. Drug development typically costs 2–3 billion USD across 13–15 years from first discovery to final regulatory approval [107]. An alternative is drug repurposing, which is strongly supported by governments and funding bodies as an efficient and effective option [108] (see [109] for detailed benefits). In addition, drug repurposing may be particularly appropriate in conditions with high oxidative stress and comorbidities [110, 111]. We propose that trimetazidine is a promising drug that can be repurposed to target mitochondrial dysfunction, inflammation and oxidative stress to treat bipolar depression.

Trimetazidine hydrochloride is an anti-ischemic agent that is widely used in coronary artery disease treatment [112]. It is a piperazine derivative with molecular formula of C1_4_H_24_Cl_2_N_2_O_3_ (1-[(2,3,4-trimethoxyphenyl) methyl] piperazine dihydrochloride) [112]. The neutral trimetazidine has very low solubility in an aqueous solution while its dihydrochloride salt form is water soluble [113]. Trimetazidine is sold as a 20 mg immediate release tablet or a 35 mg modified release tablet formulation [112, 114]. Trimetazidine is rapidly absorbed with high bioavailability, reaching peak plasma concentration of 53.6 mg/L within 1.8 h for immediate release and steady level within 24 h for modified release [112, 114].

We have identified trimetazidine as a candidate to treat bipolar depression [19] using an in silico treatment discovery model in diabetes [18] that led to a successful Phase 2 clinical trial to treat diabetes [115]. In this cross-disciplinary adaptation, human NT2-N neuronal cell cultures [116] were treated with a cocktail of first-line bipolar depression medications or vehicle to detect an overall effect of effective therapies. RNAseq was used to measure genome-wide mRNA levels to discover the gene-expression signature that best describes the overall medication effects. This gene-expression signature predicted the medication effects with power of >99%, which was then confirmed by candidate-gene assays. We then treated new NT2-N cells with positive (medication cocktail) control, negative control (vehicle), and *960 off-patent compounds* from the Prestwick library (http://www.prestwickchemical.com/libraries-screening-lib-pcl.html). Based on the changes in gene expression, a similarity score for each drug relative to the medication cocktail was calculated. We then excluded compounds that are not yet approved for human use, have potential toxicity issues, were never marketed, or were not known to cross the blood–brain barrier. After such screening, trimetazidine was identified as the most promising candidate to treat bipolar depression because it is novel in psychiatry, has an excellent safety profile, and crosses the blood–brain barrier. Using the social isolation with chronic restraint rat model, we confirmed that trimetazidine (30 mg/kg) injected once-daily for 2 weeks had an antidepressant-like effect shown by reduced immobility in the forced swim test. We also observed trimetazidine’s antidepressant effects on Flinders Sensitive Line rats that are prone to depression-like behaviors.

The main mechanism of trimetazidine is modulating mitochondrial energy production [117]. Mitochondria mainly utilize oxidation of glucose or fatty acids to produce ATP [118]. While fatty acid oxidation produces more ATP per gram, it requires more oxygen and can be slower than glucose oxidation in producing ATP, which increases risks such as hypoxia and oxidative stress to the cell [119]. Specifically, fatty acid oxidation may not keep up with required rapid ATP generation during periods of extended continuous and rapid neuronal firing, making it less suitable than glucose oxidation for brain metabolism [119]. Fortunately, inhibiting fatty acid oxidation can shift the metabolic processes to rely more on efficient glucose oxidation [118, 120]. Trimetazidine is a selective inhibitor of 3-ketoacyl-CoA thiolase, a key enzyme in fatty acid oxidation [121]. By selectively inhibiting β-oxidation of free fatty acids, trimetazidine promotes glucose oxidation and decreases oxygen consumption [121]. Trimetazidine also increases pyruvate dehydrogenase activity to decrease lactate accumulation [117]. These processes ultimately result in trimetazidine reducing intracellular calcium ion accumulation, reactive oxygen species and neutrophil infiltration to increase cellular membrane stabilization [113, 122–127].

**Fig. 2:**
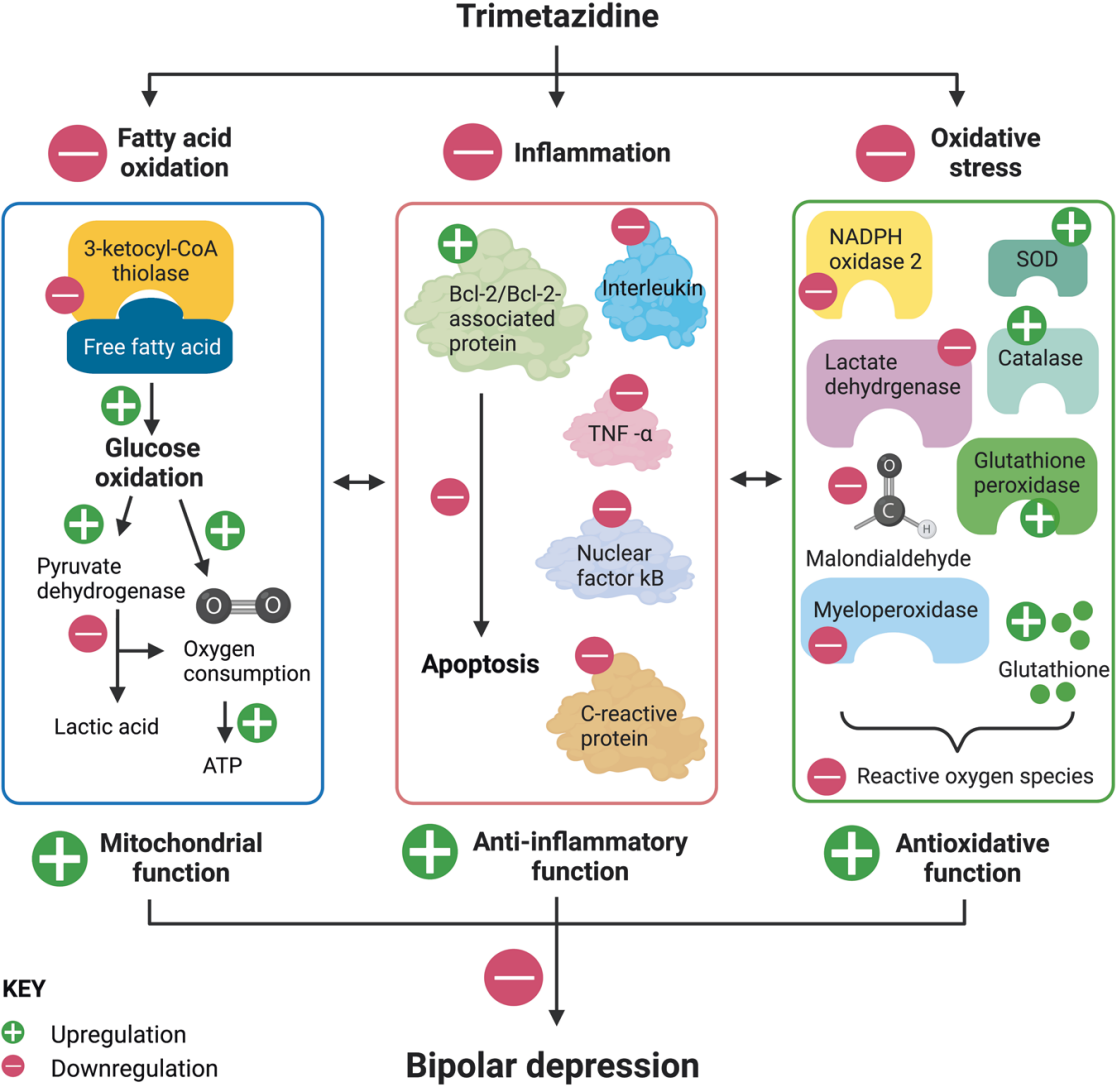
Trimetazidine is a selective inhibitor of 3-ketoacyl-CoA thiolase. Hence trimetazidine modulates mitochondrial energy production by inhibiting fatty acid oxidation to engage efficient glucose oxidation, which increases adenosine triphosphate (ATP) conversion compared to oxygen consumed. Trimetazidine also increases pyruvate dehydrogenase activity to decrease lactate accumulation. These effects ultimately reduces intracellular calcium ion accumulation and reactive oxygen species (ROS) to reduce apoptosis, inflammation and oxidative stress indicated by reduced level of biomarkers such as tumor necrosis factor alpha (TNF-α) and nicotinamide adenine dinucleotide phosphate (NADPH) oxidase 2. Trimetazidine has also been shown to increase antioxidant activity measured by increased glutathione, glutathione peroxidase, superoxide dismutase (SOD), and catalase. Taken together, accumulating preclinical and clinical evidence of trimetazidine’s regulation of mitochondrial function, anti-inflammatory and antioxidant properties strongly support its potential efficacy to reduce bipolar depression. Figure created with Biorender.com.

Trimetazidine, though introduced as an anti-anginal agent to increase metabolic efficiency when metabolic processes are compromised, is postulated to have a cytoprotective action as above [128–130]. Indeed, preclinical and clinical studies evidence beneficial effects of trimetazidine not only on mitochondrial energy metabolism but also on inflammation and oxidative stress compared to saline or vehicle [131, 132]. Such literature strongly suggests the potential of trimetazidine to address key elements of bipolar depression’s pathophysiology (Fig. 2).

## Mitochondrial and metabolic functions of trimetazidine

### Preclinical studies

Models of partial or total global ischemia followed by reperfusion in the isolated rat heart showed that trimetazidine accelerated restoration of phosphorylation, attributed to rapid energy transfer by trimetazidine [133]. This was replicated in rat kidney undergoing cold ischemia-reperfusion injury [134]. Similarly, an ex-vivo rat heart ischemia study showed that trimetazidine increased mitochondrial complex I activity, decreased oxygen consumption and free radical production thereby increasing mitochondrial integrity and maintenance of function [135].

In vivo studies also demonstrate trimetazidine’s mitochondrial effects. Lung transplantation injury was significantly prevented by an injection of trimetazidine (5 mg/kg) to donor and receiver, which caused significantly higher ATP levels and better oxygenation [136]. In rats, gavage of trimetazidine (10 mg/kg/day) for 7 days prior to induction of acute myocardial ischemia preserved the mitochondrial structure, improved respiratory control ratio and mitochondrial complex I activity, as well as mitochondrial biosynthesis and fusion [137].

Trimetazidine improves mitochondrial function beyond ischemic damage. Intraperitoneal injections of trimetazidine (25 mg/kg/day for 5 days) in rats before seizure induction prevented apoptosis of hippocampal neurons [138]. Trimetazidine increased serum and platelet levels of serotonin when administered to rats with induced myocardial infarction and depression [139]. However, brain serotonin levels were significantly decreased. The authors hypothesized that this was due to trimetazidine affecting the brain tissue cell metabolism, and potentially transporting serotonin from brain tissue into the peripheral nervous system. Cultured rat myocytes challenged with palmitate showed decreased mitochondrial ATP levels, oxygen consumption rate, mitochondrial volume, and increased mitochondria per cell indicating increased mitochondrial fission [140]. All of these processes were reversed by pre-treatment with trimetazidine [140]. Diabetes-associated cardiomyopathy in rats was rescued by oral gavage of trimetazidine for 8 weeks (30 mg/kg/day), which also decreased insulin resistance [141].

While trimetazidine’s ability to increase mitochondrial function is clear, these findings generally did not assess trimetazidine’s effects on healthy tissue. Therefore, an important question remains. Does trimetazidine push mitochondrial processes over and above healthy mitochondrial function? Fantini et al. showed that in cultured rat ventricular myocytes, trimetazidine’s beneficial effects were only observed when cells were undergoing hypoxia and not during normoxia, highlighting trimetazidine as a metabolic regulator that would not increase mitochondrial function in healthy states [123]. Similarly, trimetazidine treatment (0.5 mg/kg) was protective of mouse myocytes in an ischemia/reperfusion injury via anti-apoptotic pathway without affecting healthy controls [142]. One study in healthy rat brain mitochondria also did not observe any direct effect of trimetazidine on mitochondrial permeability transition [143].

### Clinical studies

Patients with past myocardial infarction who underwent perfusion imaging and revascularization showed evidence of improved mitochondrial oxidative metabolism from a single tablet of trimetazidine (60 mg) compared to placebo [144]. Three months of trimetazidine (70 mg/day) in patients with cardiomyopathy decreased the myocardial β-oxidation rate compared to placebo, implying its ability to shift mitochondrial processes to glucose oxidation [145]. Two periods of 90 days of trimetazidine (60 mg/day) also increased cardiac phosphocreatine/ATP ratio in nine patients (but three patients showed a decrease), which indicated improved mitochondrial energetics [146].

## Anti-inflammatory functions of trimetazidine

### Preclinical studies

Neutrophils are a key part of the immune system as the major type of white blood cells [147]. Neutrophil increase is a reliable marker of inflammation [148, 149]. Inflammation following myocardial infarction of anaesthetized rabbit heart was significantly protected by an acute pre-infarction intravenous trimetazidine infusion, with lowered neutrophils [150]. Chronic trimetazidine injection (3 mg/kg/day for 1 week) in rats had similar outcomes [151]. In vitro human neutrophils activated by formyl-methonyl-leucyl-peptide were also attenuated by trimetazidine [151]. Interestingly, trimetazidine appears to only reduce neutrophils when levels become too high. In mouse sepsis and endotoxemia, trimetazidine (60 mg/kg) promoted neutrophil recruitment to the heart tissue and alleviated myocardial dysfunction [152].

Lipopolysaccharide injection induces robust inflammation, and trimetazidine (60 mg/kg/day for 3 days) protected against lipopolysaccharide-induced myocardial dysfunction and apoptosis by inhibiting macrophage pro-inflammatory cytokines [153]. In fact, inflammation often resolves with apoptosis [154], and much of the evidence for anti-inflammatory actions of trimetazidine comes from demonstrations in apoptosis and cell survival. For example, oral gavage of trimetazidine for 8 weeks (30 mg/kg/day) reduced cardiac apoptosis in diabetic rats [141]. Inhibition of cardiac apoptosis by trimetazidine (2.5 mg/kg acute pre-treatment) was replicated in swine with myocardial infarction [155]. In a similar mini pig microembolization model, trimetazidine pre-treatment (2.5 mg/kg) reduced myocardial damage by inhibiting the pro-inflammatory programmed cell death/nuclear factor kB/TNF-α pathway [156]. In a neonatal rat in vitro cardiomyocyte hypoxia/reoxygenation study, pre-treatment with trimetazidine reduced apoptosis and inflammation [157]. These anti-inflammatory properties of trimetazidine were replicated in a mouse sunitinib-induced cardiotoxicity model [158].

Trimetazidine was also shown to reduce inflammatory markers in other organs beyond the heart and blood. In an ischemic pig kidney, 5 or 10 mg/kg of trimetazidine given intravenously significantly reduced CD4+ lymphocytes [159]. When cultured murine skeletal muscle cells were atrophied by the pro-inflammatory cytokine TNF-α, not only did trimetazidine significantly reverse the reduction in myotube size, it also increased myosin heavy chain expression and induced hypertrophy [160]. These studies clearly highlight trimetazidine as an anti-inflammatory medication peripherally. Trimetazidine is lipophilic and crosses the blood-brain-barrier [112], hence it is highly likely to exert anti-inflammatory effects also in the brain. At least one study showed that an acute injection of trimetazidine (10 mg/kg) reduced hippocampal inflammation measured by TNF-α and IL-1β while protecting against seizure and associated cognitive impairments in diabetic and epileptic rats [161]. These effects were correlated with increased ATP/adenosine diphosphate ratio [161].

### Clinical studies

Trimetazidine has been clinically studied for its effects on several direct and indirect markers of inflammatory responses. Oral trimetazidine (60 mg/day) taken for three days prior to percutaneous transluminal coronary angioplasty reduced CRP and nitrite levels both at pre- and post-angioplasty and TNF-α at post-angioplasty compared to no treatment [162]. Similarly, oral trimetazidine (20 mg three times a day) among volunteers with diabetes and idiopathic dilated cardiomyopathy maintained CRP levels stable over 6 months compared to a placebo control that showed increased CRP [163]. Bobescu and colleagues (2021) conducted a large study involving 570 patients with inadequately treated symptoms of coronary artery disease to examine the effect of trimetazidine (70 mg/day). They observed trimetazidine significantly reduced CRP levels at 6 months from treatment compared to no treatment [117].

In participants receiving coronary artery bypass grafting, 12–15 days of oral trimetazidine (60 mg/day) significantly reduced IL-6 levels compared to placebo treatment at baseline, 5 mins after aortic unclamping, 12 and 24 h after surgery [164]. When Shao et al. examined trimetazidine (60 mg/day) alone and in combination with coenzyme Q10 in acute viral myocarditis, both groups receiving trimetazidine showed decreased pro-inflammatory makers such as TNF-α and IL-8 at 2 weeks of treatment compared with baseline [165]. In a randomized study where trimetazidine (35 mg/day) was prescribed for 4 days during elective coronary scaffold implantation, the trimetazidine group showed reduced IL-6 [166]. In people with stable refractory angina, adjunctive trimetazidine (70 mg/kg/day) significantly enhanced external counter pulsation intervention by decreasing inflammatory markers such as IL-1β [167].

## Antioxidant functions of trimetazidine

### Preclinical studies

In vitro evidence supports trimetazidine as an antioxidant. Cisplatin induced cardiotoxicity in rat myocytes benefited from trimetazidine, which reduced ROS and the oxidative stress product malondialdehyde while increasing antioxidant SOD and catalase [168]. In rat embryonic myocytes with hypoxia/reoxygenation, trimetazidine pre-treatment mitigated the increase in oxidative stress proteins such as lactate dehydrogenase and ROS [169]. In cultured human endothelial progenitor cells, trimetazidine protected against hydrogen peroxide induced oxidative stress by increasing SOD and reducing malondialdehyde [170].

There is also in vivo evidence, with trimetazidine (5 mg/kg) pre-treatment attenuating superoxide levels in the rat heart following ischemia/reperfusion [171]. In rats with type 2 diabetic cardiomyopathy, trimetazidine (10 mg/kg/day for 11 weeks) alleviated diabetes induced structural and functional changes of the heart by inhibiting oxidative stress [172]. Further, acute trimetazidine injection (5 or 10 mg/kg) significantly decreased ROS in cardiomyocytes in rats with myocardial infarction [173]. In atherosclerotic rats, trimetazidine (30 mg/kg/day for 12 weeks) prevented ROS upregulation, and restored antioxidant SOD levels while reducing oxidized low-density lipoprotein and malondialdehyde [169]. Trimetazidine (10 mg/kg/day for 2 weeks) reduced peripheral blood oxidative stress caused by amikacin, a widely prescribed antibiotic that can produce ototoxic effects that can cause damage to the cochlea [174]. Trimetazidine (5 or 25 mg/kg single injection) restored SOD levels in the brain after it was reduced following cerebral ischemia-reperfusion injury [175]. Trimetazidine (10 or 20 mg/kg/day for up to 5 weeks) also significantly alleviated pentylenetetrazole-induced seizure in mice while reducing lipid peroxidation and increasing glutathione levels in the brain [176].

In ischemia/reperfusion injured rat intestine, an infusion of intravenous trimetazidine (3 mg/kg) lowered malondialdehyde and myeloperoxidase, which is a pro-oxidative enzyme that catalyzes the formation of ROS [177]. In renal tissue, acute or chronic systemic injections of trimetazidine have been shown to reduce thiobarbituric acid reactive substances in immunosuppressant-induced renal dysfunction [178] and prevent SOD, glutathione peroxidase, catalase or glutathione decrease in ischemia/reperfusion injury in rats [179, 180]. Similar findings were observed in an ischemia/reperfusion rat forebrain injury, in which chronic trimetazidine (alone or with progesterone) alleviated SOD and glutathione decrease while preventing malondialdehyde and lipid peroxidase increase [181]. Also in the rat brain, pre-treatment injection of trimetazidine (25 mg/kg/day for 7 days) prevented oxidative changes measured by SOD, catalase and malondialdehyde in a model of a sporadic type of Alzheimer’s disease [182]. The latter findings confirm that trimetazidine can cross the blood-brain-barrier to reduce oxidative stress in the brain.

Lastly, trimetazidine can alleviate oxidative stress following lifestyle-related injuries. Oral trimetazidine (6 mg/kg/day for up to 42 days) in rats was able to prevent the increase in malondialdehyde and nitric oxide and decrease in glutathione in the crushed sciatic nerve [183]. Eight weeks of high fat diet in mice decreased insulin sensitivity and manganese-dependent SOD activity while increasing malondialdehyde, all of which was prevented by co-administration of trimetazidine (10 mg/kg/day, intragastric) [184]. In that study, the effect size of trimetazidine on reducing oxidative stress was larger than daily exercise intervention, showing its potential as a powerful antioxidant.

### Clinical studies

Two weeks of trimetazidine (60 mg/day for 2 weeks) alone or in combination with coenzyme Q10 in people with acute viral myocarditis increased SOD and glutathione while reducing malondialdehyde compared to baseline [165]. Trimetazidine (60 mg/day for 6 months) was also effective in reducing malondialdehyde compared to baseline among a group of patients with end stage renal disease on hemodialysis and continuous peritoneal ambulatory dialysis [185].

## Conclusions and looking ahead

Bipolar depression is different from major depressive disorder in its ontogeny and clinical characteristics [11, 186]. Their biological differences are highlighted by the fact that antidepressant monotherapy is not recommended to treat bipolar depression [7, 13]. In this review, three major biological processes associated with bipolar depression were highlighted. Overall, evidence for mitochondrial dysfunction, inflammation and oxidative stress in bipolar depression is consistent with many replicated findings across tissue types, molecular assays, and ethnicities [187, 188]. A particularly compelling hypothesis is that bipolar depression is a state of decreased mitochondrial energy generation, which may be overcompensated by increased mitochondrial energy generation in mania [20–25]. Trimetazidine’s main activity in boosting mitochondrial energy generation only when mitochondrial function is reduced, while also targeting inflammation and oxidative stress that occurs in both depression and mania in bipolar disorder makes it a promising novel pharmacotherapy candidate to be tested in clinical trials. Should such trials yield positive outcomes, it can be rapidly translated into clinical care to treat bipolar depression due to its availability, low cost, safety, and tolerability. A randomized, double-bind, placebo-controlled trial of chronic trimetazidine as an adjunct therapy in >6000 patients who had undergone successful percutaneous coronary intervention at 365 centers in 27 countries across Europe, North Africa, Asia, and South America showed strong evidence for the safety and tolerability of trimetazidine compared to placebo at 27.5 months of chronic daily administration [189]. Such a safety profile is better than existing first-line treatments for bipolar depression [7]. Taken together, this review provides a rationale for the use of trimetazidine as a promising repurposing candidate to treat bipolar depression.
